# The Gibberellin Producer *Fusarium fujikuroi*: Methods and Technologies in the Current Toolkit

**DOI:** 10.3389/fbioe.2020.00232

**Published:** 2020-03-27

**Authors:** Yu-Ke Cen, Jian-Guang Lin, You-Liang Wang, Jun-You Wang, Zhi-Qiang Liu, Yu-Guo Zheng

**Affiliations:** ^1^Key Laboratory of Bioorganic Synthesis of Zhejiang Province, College of Biotechnology and Bioengineering, Zhejiang University of Technology, Hangzhou, China; ^2^Engineering Research Center of Bioconversion and Biopurification of Ministry of Education, Zhejiang University of Technology, Hangzhou, China

**Keywords:** *Fusarium fujikuroi*, genetic engineering, fermentation, tools, gibberellic acid, CRISPR-cas

## Abstract

In recent years, there has been a noticeable increase in research interests on the *Fusarium* species, which includes prevalent plant pathogens and human pathogens, common microbial food contaminants and industrial microbes. Taken the advantage of gibberellin synthesis, *Fusarium fujikuroi* succeed in being a prevalent plant pathogen. At the meanwhile, *F. fujikuroi* was utilized for industrial production of gibberellins, a group of extensively applied phytohormone. *F. fujikuroi* has been known for its outstanding performance in gibberellin production for almost 100 years. Research activities relate to this species has lasted for a very long period. The slow development in biological investigation of *F. fujikuroi* is largely due to the lack of efficient research technologies and molecular tools. During the past decade, technologies to analyze the molecular basis of host-pathogen interactions and metabolic regulations have been developed rapidly, especially on the aspects of genetic manipulation. At the meanwhile, the industrial fermentation technologies kept sustained development. In this article, we reviewed the currently available research tools/methods for *F. fujikuroi* research, focusing on the topics about genetic engineering and gibberellin production.

## Introduction to *F. fujikuroi*

*F. fujikuroi* is a prevalent plant pathogen, which causes the bakanae disease of the rice plant. The sick plants grew inordinately long, and eventually felled off and died. This phytopathogen was latterly found causing devastating disease in many other economically important plants, including maize, sugarcane, wheat, asparagus etc. In the early 20th century, scientists from Japan, United Kingdom, and United States isolated the active compounds, gibberellic acids (GAs), which was also isolated later from the higher plants (Mitchell et al., 1951). Since then, differentially structured GAs were isolated, and GAs became a large family of structurally identical diterpenoids with 136 known isoforms, of which some are active plant hormones, including GA_1_, GA_3_, GA_4_, and GA_7_ (Blake et al., 2000; Bomke and Tudzynski, 2009; Rodrigues et al., 2012). GAs are now classified as one of the five major types of phytohormones, namely the auxins, cytokinins, gibberellins, abscisic acid, and ethylene. The use of ppm (parts per million, mg/l) levels of GAs may result in physiological effects such as elimination of dormancy in seeds, acceleration of seed germination, improvement in crop yield, promotion of fruit setting, overcoming of dwarfism etc. GAs have been widely applied to improve the quality and quantity of fruit, crop and omamental plants. Although GAs are present extensively in plants, fungi and bacteria, *F. fujikuroi* is the only organism being applied for industrial production of GAs, as it shows excellent productivity. Taking the product GA_3_, a representative GA product of *F. fujikuroi*, as an example, the yield in industrial submerged fermentation (SMF) has reached more than 2g/L after 7 days fermentation, while in solid state fermentations (SSF), its yield reached 7 g/kg support or even higher after 9 days fermentation. These values are much higher than the reported yield of other microbes. Besides, many other valuable secondary metabolites were also discovered to be produced by *F. fujikuroi*, indicating the potential of *F. fujikuroi* to be apply for production of other chemicals (Janevska and Tudzynski, 2018).

GA induced signal transduction is very complicated in plants, while overdose of GAs may result in plant death (Eckardt, 2002). As a phytopathogen, fusaria can be loosely classified as hemibiotrophs. Upon infection with *F. fujikuroi*, the plant becomes sick/weak, and gets subsequently more easy to be invaded, which could be largely due to the contribution of GAs secretion. Research from Wiemann et al. showed that the infected rice plant experienced dramatically increased invasive fungal growth of a GA-secreting wild type *F. fujikuroi* when compared to its GA-deficient mutant (Wiemann et al., 2013). At the meanwhile, the enlarged plant body by the abnormal elongation might also provide the pathogen additional space and nutrients. Eventually, the infection turns to the stage of killing and consuming the host body, while the fusaria become necrotrophic in this stage (Ma et al., 2013).

It would be interesting to reveal the underlying mechanism of the virulence factors of *F. fujikuroi*, which may help to discover the potential antifungal targets or to develop a strain that are non-pathogenic and safe for the agricultural environment. Currently, we still lack the systemic knowledge to control the pathogenesis of *F. fujikuroi*. The virulence/pathogenicity genes of some *Fusarium* species has been characterized and summarized in some review articles (Michielse and Rep, 2009; Walter et al., 2010; Kazan et al., 2012). The virulence linked host-pathogen interaction is a very complicated process with a massive amount of genes and regulators involved. Based on the infection strategies, Ma et al. classified the virulence genes into two types. The genes of the first class were named as the basic pathogenicity genes, which is universal in the *Fusarium* genus and shared with many other pathogens. Genes of mitogen-activated protein kinase (MAPK) signaling pathways, Ras proteins (small GTPases), G-protein signaling components and cAMP pathways etc. are involved in this class. These genes usually correlate also globally with the cell fitness. The genes in the second class were named as the specialized pathogenicity genes, which is usually specific to a *Fusarium* species on specific hosts (Ma et al., 2013). GAs production is apparently a key virulence factor of *F. fujikuroi* and requires a set of specialized pathogenicity genes. However, GAs production is not essential to the virulence. Deletion of the entire GAs gene cluster could neither impair the host-cell colonization nor abolish the invasive growth completely in a rice-root infection experiment (Wiemann et al., 2013). Besides GAs, *F. fujikuroi* synthesizes a large amount of other metabolites, of which many are toxic compounds. In addition, many secreted enzymes may also help the fusaria to penetrate the cell wall and ultimately invade the plant. Bashyal et al. analyzed the *F. fujikuroi* genome and predicted that there were 1194 secretory proteins, of which 38% proteins might relate to the virulence. Moreover, out of secretory proteins, 5% were polysaccharide lyases, 7% were glycosyl transferases, 20% were carbohydrate esterases, and 41% were glycosyl hydrolases (Bashyal et al., 2017). It is interesting to exploit further experimentally the specialized pathogenicity genes, especially some secreted cell wall degrading enzymes and mycotoxins in *F. fujikuroi* (Desmond et al., 2008).

Beside the gibberellin-producing fusaria, the helminthosporol-producing *Helminthosporium sativum* was also focused. Helminthosporol is a natural sesquiterpenoid that is able to induce GAs like bioactivity (Miyazaki et al., 2017) and cause seedling blight and root rot in some plants (Pringle, 1976). However, far less is known about the biosynthesis of helminthosporol and the biology of *H. sativum*, when compared to GAs and *F. fujikuroi*. Compared to helminthosporol, GAs are prevalently present in the high plants in nature, thus have a broader application, whereas helminthosporol is a plant growth regulator that synthesized by the microorganism. Besides, although helminthosporol and its analogs helminthosporal and helminthosporic acid, have GA-like activity in some plants, they act less efficient or differentially in many experiments. For instance, helminthosporol and helminthosporic acid work less efficiently than GAs in reversing 2-chloroethyl-trimethylammonium chloride induced dwarfing on the hypocotyls of lettuce seedlings and in stimulating sugar release from de-embryonated barley (Briggs, 1966). The GA biosynthetic inhibitor prohexadione did not inhibit the shoot elongation caused helminthosporic acid. *H. sativum* infected wheat was not elongated, because helminthosporol has no GA activity in wheat. *H. sativum* did not infect the rice plant as a host, although helminthosporol may promote rice seedlings (Miyazaki et al., 2017).

A prerequisite to characterize the molecular biology is having efficient analysis tools. After a number of *F. fujikuroi* genome sequences are available (Jeong et al., 2013; Wiemann et al., 2013; Bashyal et al., 2017; Niehaus et al., 2017), it is more eager than ever to develop molecular tools. However, unlike many other fungi, such as the baker’s yeast and some phylogenetically close *Aspergillus* spp., *F. fujikuroi* is critically short of molecular tools. Lacking of handy molecular tools has become the major obstacle for the development of the *F. fujikuroi* research, especially in the aspect of genetic modification of this fungus. Genetic manipulation is difficult in this microorganism, which is due to poor protoplast formation, inefficient transformation, low homologous recombination (HR) rate etc. In this review, currently applying methods and tools, including the methods to identify *Fusarium* species, plant infection assays, sexual cross method, promoters for gene expression, plasmid toolbox, protocol of protoplast preparation, transformation technologies, genome editing strategies, RNA-mediated gene silencing assay, protein fluorescent tags, methods of biomass quantification, gibberellin fermentation technologies and strategies of strain improvement, have been reviewed. We summarized the currently using materials and techniques for *F. fujikuroi* research, providing a perspective in the development of molecular tools for this industrial and agricultural important fungus.

## Identification of *Fusarium* Species

The *Fusarium* species are ubiquitous in nature, and are extensively distributed in soil, plants and various organic substances. Identification of the *Fusarium* species becomes crucial for agricultural application, healthcare purpose and scientific investigation. To date, hundreds of *Fusarium* genome sequences have been deposited in the database. These genome sequences can be used as efficient and essential tools for identification of *Fusarium* species, gene/enzyme mining, evolutionary, and phylogenetic analysis etc. (Ward et al., 2002; O’Donnell et al., 2013; Bashyal et al., 2017). There are currently 68 *Fusarium* species with their genome sequences available in the NCBI (National Center of Biotechnology Information, United States) database. Among them, *Fusarium oxysporum*, *F. fujikuroi*, *Fusarium proliferatum* and *Fusarium graminearum* are the best focused four species. The numbers of their genome assemblies in the database are 222, 18, 13, and 11 respectively. These *Fusarium* species are all prevalent phytopathogens and economically very important, thus are also better studied and with more molecular tools available when compared with the other species in the *Fusarium* genus. The *Fusarium fujikuroi* species complex (FFCS), previously known as *Gibberella fujikuroi* species complex, contains about 50–100 phylogenetically close *Fusarium* species, of which *F. fujikuroi*, *Fusarium proliferatum*, and *Fusarium verticillioides* are best studied. The taxonomy of FFSC was based on the evolutionary, biological and morphological species concepts (Kvas et al., 2009; Summerell et al., 2010), whereas the modern biology also employs sequencing data.

Conventionally, the identification of microorganisms is mainly based on the morphology. The morphological identification is generally based on the macroscopic and microscopic characteristics. The macroscopic characteristics include the colony appearance, pigmentations and growth rates. The FFSC cells present usually as white to dark purple cottony aerial mycelium. The microscopic characteristics include the microscopic observation of the macroconidia, microconidia, chlamydospores, the mode of microconidial formations etc. At the moment, the most recent and systemically documented guide for morphological characterization of the *Fusarium* species was contributed by Leslie and Summerell (Leslie and Summerell, 2006). However, the morphological identification is time consuming and could easily result in misidentifications, especially for the phylogenetically close species (Hsuan et al., 2011; Raja et al., 2017). Although it might be problematic to use morphology alone, this method is still helpful in practice and is frequently used now in combination with other molecular means.

The MALDI-TOF MS (Matrix-assisted laser desorption/ionization time-of-flight mass spectrometry) assay is an advanced tool for rapid and accurate identification of microorganisms. This technique has been widely applied to identify bacteria, yeasts and other fungi (Pinto et al., 2011; Mesureur et al., 2018; Pauker et al., 2018; Quero et al., 2019; Kim et al., 2019), especially for the identification of the human pathogens, whereas might be relatively less popular in the plant pathogen research at this moment. However, a broader application can be foresee in the near future based on its excellent accuracy and efficiency, and fast development of analyzing equipment. Briefly, this assay is carried out based on the mass spectral readout of the molecular mass from the ionized protein mixture. Thus, each cell culture may result in a very specific mass spectral pattern, which can be taken as the unique fingerprint to identify a microorganism from the very closely related species (Huschek and Witzel, 2019). To be taken as an identification tool, a database of such mass spectral patterns has to be established beforehand. A MALDI-TOF MS database has been established with 24 reference strains for identification of mainly the clinical isolates belonging to the FFSC. It was reported that 93.6% of the isolates can be correctly identified to the species level (Al-Hatmi et al., 2015). Recently, Wigmann et al. expanded the database (Wigmann et al., 2019). In their work, MALDI-TOF MS was carried out for 49 species from the species complex, taking the sequencing data of the translation elongation factor 1 α (*TEF1*α) gene as the reference. The MALDI-TOF MS fingerprints were then taken as a database to screen over 80 isolates from the FFSC, and resulted in a high correct-identification-rate of 94.61%.

PCR based cell identification is another type of rapid, accurate and cost effective method to identify the microorganisms. Unlike the MALDI-TOF MS method, the PCR based methods require only the routine facilities in a molecular lab. The PCR based methods have been developed for the *Fusarium* species identification since many years ago, whereas without a standardized protocol. Usually, different genomic loci were targeted, and ended up with diverse forms of results. The galactose oxidase gene *gaoA* was taken as the PCR target to identify the *Fusarium* species, as the gene region has very low homology among the fungi (Niessen and Vogel, 1997; de Biazio et al., 2008). The internal transcribed spacer (ITS) regions have been successfully used to identify some closely related fungi. The ITS regions of the conserved rDNA have been successfully used to identify some *Fusarium* species (Abd-Elsalam et al., 2003; Lacmanova et al., 2009). The *TEF1*α gene is usually a single copy gene in the *Fusarium* genus, and is frequently employed for species identification, as it also presents a high level of sequence polymorphism in different species. Other genes such as the β-tubulin, RNA polymerase II (*RPB2*), nitrate reductase, phosphate permase, and the mitochondrial small subunit were also targeted for PCR identification. However, for a better resolution, a multi-locus sequence typing (MLST) method should be used by targeting multiple genes. Usually, at least three gene loci were taken for such identifications (Baayen et al., 2000; O’Donnell et al., 2000; Skovgaard et al., 2001). As an example, Ke et al. (2016) identified the *Fusarium* species by PCR of ITS, *RPB2* and *TEF1*α. Faria et al. (2012) developed a multiplex PCR method after testing 6 pairs of primers targeting different genes/genomic DNA of different *Fusarium* species. The failure/success of PCR amplification, using different pairs of primers, was counted to determine the belonging of a specific *Fusarium* species (Faria et al., 2012). Recently, a *TEF1*α LAMP (Loop-Mediated Isothermal Amplification) based identification method has been developed for detection of the seedborne *F. fujikuroi* and *Magnaporthe oryzae* in rice seeds. Four independent *F. fujikuroi* isolates were tested taking their serially diluted DNA samples as the amplification templates. Based on the time-to-positive of the LAMP assay, the authors claimed that this assay showed a detection sensitivity/limit of 100–999 fg (vary among different isolates) of *F. fujikuroi* DNA (Ortega et al., 2018).

## Plant Infection Assays

**FIGURE 1:**
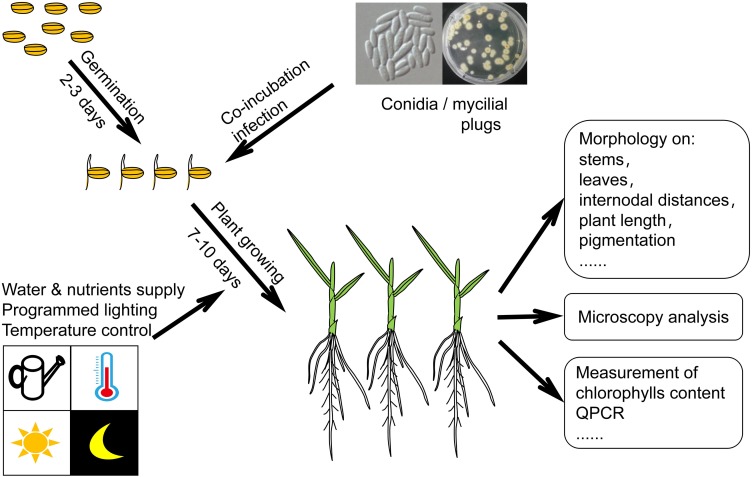
Schematic diagram of a *F. fujikuroi*-rice plant infection assay.

Although *F. fujikuroi* may invade many plants, the rice plant is a preferred host. The ability to cause rice bakanae disease has become the hallmark of the microorganism *F. fujikuroi*. Thus, the rice plant was frequently chosen as the host to investigate the virulence of *F. fujikuroi*. Wiemann et al. investigated *F. fujikuroi* virulence that linked to a velvet-like protein complex using a rice plant infection assay. In their experiment, the husks removed rice seeds were incubated for 3 days in agar gel for germination and co-incubated subsequently with 5 mm diameter *F. fujikuroi* mycelial plugs in Vermiculite filled test tubes. The infected plants were grown for another 7 days, supplying with water and nutrients. The germination period and growth period were both implemented at 28°C under a 12 h light – 12 h dark cycle. Finally, the bakanae symptoms such as chlorotic stems and leaves were observed and documented (Wiemann et al., 2010). Adam et al. infected the rice plants using conidia samples of different *F. fujikuroi* strains. In their experiment, the rice seeds were germinated for 2 days for seedlings with developed shoots/roots length of 1–2 mm. A fixed amount of conidia were co-inoculated then with the prepared seedlings for infection. The infected plants were grown for another 10 days with the programmed lighting and nutrient supply. Finally, the plant length and internodal distances were recorded, while the paler pigmentation of the bakanae disease was characterized and verified by measuring their content ratio of chlorophylls/carotenoids (Adam et al., 2018). Similar to the previously described experiment, the whole assay took around 2 weeks to evaluate the systemic *F. fujikuroi* infection of the rice plant *in vivo* (see Figure 1). A rice/maize root infection assay was carried out by Wiemann et al. to evaluate the pathogenicity of GAs production. The pathogens were inoculated by co-cultivation with the geminated rice and maize seeds. Fixed temperature and humidity, and programmed lighting cycles (differentially for rice and maize) were supplied to the infected seedlings in agar gel support. After 10 days of growth the root samples were collected for visualization of invasive growth of the corresponding pathogen by fluorescence microscopy, and the penetration events were quantified. At the meanwhile, pathogen spores (10^4^/ml) were collected for measurement of relevant mRNA by RT-PCR (Wiemann et al., 2013). On the basis of the described infection assays, mostly the rice seedlings were preferred to be chosen as the host plant. Different fungal samples, such as mycelia and conidia, can be used to infect the seedlings, while different methods are available to characterize the pathogenicity/virulence (see Figure 1).

## Sexual Cross

Crossing is always a powerful method to combine genotypes, exchange genetic materials and obtain large-scale mutations. Studt et al. implemented a sexual cross between a pair of mating partners, C1995 and IMI 58289, two well-studied laboratory strains with opposite mating types, Mat-2 and Mat-1, respectively. With the experiment, they pinpointed that a newly found gene *FSR1* was involved in perithecial pigmentation in *F. fujikuroi* (Studt et al., 2012). The crossing experiment was carried out using a carrot agar medium, which has been reported by Klittich et al. previously (Klittich and Leslie, 1988). The crossing protocol has been well documented by Zakaria et al. (2011). Briefly, in their experiment the female parent and male parent were inoculated in the carrot medium and complete medium respectively. After 7 days growth at 25°C, the mycelium from the male parent was harvest and suspended in Tween 60 for spore suspension, which was subsequently spread into the mycelium of the female parent on a carrot agar plate. The carrot agar plate was then incubated at 27°C for a few weeks until the perithecia were produced. The *F. fujikuroi* species complex was divided into many biological species, designated as mating populations A to J. *F. fujikuroi* is the mating population C. Generally, *F. fujikuroi* is heterothallic, and should be readily crossed in the laboratory. However, sexual fertility varies from strain to strain, making the sexual crosses not always successful (Zakaria et al., 2011).

## Promoters

**FIGURE 2:**
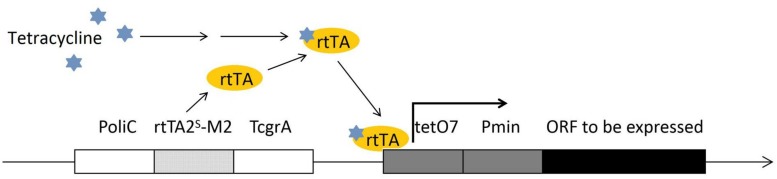
The construct of Tet-on promoter for conditional expression in *F. fujikuroi*. The promoter region is composed of a tetracycline-dependent transactivator rtTA2^S^-M2 (on the left of the construct, encodes rtTA protein) and an rtTA protein driven operator tetO7. The tetracycline activated rtTA protein is capable to bind the tetO7 operator and induce the targeted gene expression.

Selection of a proper promoter is crucial in genetic engineering. Usually, based on the research purpose and the gene to be expressed, a native promoter, a constitutively expressing promoter or an inducible promoter can be used. In *F. fujikuroi*, the most frequently used promoters, such as the *gpdA* (Michielse et al., 2014) *oliC* and *trpC* (Rosler et al., 2016) promoters, are originated from the *Aspergillus* spp. and provide very strong expression. The native strong *glnA* promoter can be induced under the nitrogen starvation condition, while can be repressed under the addition of NH_4_NO_3_ or glutamine (Teichert et al., 2004). The transcriptional regulation of *glnA* is on the basis of the transcription factor AreA, which is extensively involved in regulation of a wide range of metabolism pathways. Thus, nitrogen starvation/induction is closely linked to the synthesis of many important secondary metabolites, suggesting that potential conflict between the *glnA* expression and the research purpose has to be taken care of before the *glnA* promoter is chosen. The *glnA* promoter has been used for conditional expression of a gene in *F. fujikuroi* (Teichert et al., 2006). The *alcA* promoter is another strong inducible promoter that has been successfully applied in *F. fujikuroi* research (Teichert et al., 2006). The *alcA* promoter driven gene expression can be well induced by 1% (V/V) ethanol and repressed by 2% (W/V) glucose in *F. fujikuroi*. The *alcA* promoter is originated from *Aspergillus nidulans*. *alcA* and two other genes, *alcR* and *aldA*, are the genes of ethanol regulon, and are all transcriptional regulated by CREA and ALCR proteins (Mathieu and Felenbok, 1994). The *glnA* and *alcA* promoters are both strong promoters. Besides these, a Tet-on system has been developed for *F. fujikuroi* (Janevska et al., 2017). This Tet-on system was established based on the adaptation of a Tet-on system of *Aspergillus niger* (Meyer et al., 2011). This expression construct was composed by an *oliC* promoter, a tetracycline-dependent transactivator rtTA2^S^-M2, an *A. fumigatus* terminator *TcgrA*, and an rtTA2S-M2-dependent promoter *tetO7*:Pmin (see Figure 2). The constructed Tet-on promoter has been shown to successfully activate a silent gene cluster in *F. fujikuroi* by adding 50 μl/ml doxycycline.

## Plasmid Toolbox

Plasmids have formed an essential part in molecular biology and genetic manipulation. However, compared to the model organisms, such as *Saccharomyces cerevisiae*, which has the most diverse plasmids, *F. fujikuroi* has almost no specific plasmid for use. In *F. fujikuroi*, the currently working plasmids are generally integrative plasmids that originated from the plasmid toolbox of the *Aspergillus* species. An *A. nidulans* DNA fragment AMA1, which enables autonomous replication (AR) of plasmids in some *Aspergillus* species, such as *A. nidulans*, *A. niger*, and *Aspergullus oryzae* (Gems et al., 1991), has been tested in *F. fujikuroi*. The transformation efficiency increased over two times by using the AMA1 integrated AR plasmid in comparison with the backbone plasmid. However, this plasmid showed to be very instable inside the cells. After 10–19 days incubation in non-selective medium, only 8–44% of the cells still contain the selection marker (Bruckner et al., 1992). In addition, the southern blot test of the cells transformed with this plasmid gave very weak bands when compared to the cells transformed with the backbone plasmid, although the total amount of DNA used for this assay is equal for both conditions, suggesting that transformation of AMA1 plasmid resulted in a low copy number of the plasmid maintaining in the cells.

It would be interesting to test a centromeric plasmid based on this AR construct. In yeast, a centromeric plasmid usually works as a small chromosome, with one or two copies in each cell, which provides a stable expression profile. Some years ago, yeast centromere *CEN11* had been tested with an plasmid in *A. nidulans* (Boylan et al., 1986). However, this yeast centromere seems to be unfunctional in *A. nidulans*, since it has no effect in plasmid stability, and does not prevent chromosomal integration of the vector. Thus, to construct a centromeric plasmid, it might be necessary to test a native centromere of *F. fujikuroi*. In fact, several *Fusarium* species have been known to contain dispensable mini-chromosomes. These mini-chromosomes stay independently from the other chromosomes, can somehow communicate between neighborhood cells and contribute to the pathogenicity (Nagy et al., 1995; Ma et al., 2010; Ma and Xu, 2019; Peng et al., 2019). However, little is known about the entity of these mini-chromosomes. It would be very interesting to exploit how these chromosomes are utilized, manage to replicate, and are selectively present in the host fungi.

The selective markers are essential for the screening of plasmid transformations. In *F. fujikuroi*, the choices of selection markers are very limited. Drug resistance markers, as the representatives the nourseothricin resistance marker *nat1* (Teichert et al., 2006; Bomke et al., 2008; Janevska et al., 2017), hygromycin resistance marker *hph* (Studt et al., 2013a; Wagner et al., 2013) and geneticin (G418) resistance marker *nptII* (Castrillo et al., 2013), were the most frequently used selective markers in *F. fujikuroi*. Nutrition selection markers were hardly seen to be used in this microorganism, for instance the use of auxotrophic complementary marker genes, which could be due to the lack of constructed auxotrophic strains. Sanchez-Fernandez et al. (1991) mutated the nitrate reductase gene *niaD* in *F. fujikuroi*, and developed a selection system employing a complementary *niaD* gene of *A. niger*. With this system, the transformants were screened for the ability to utilize nitrate as the sole nitrogen source. The *A. niger niaD* gene was subsequently replaced by a native *niaD* gene of *F. fujikuroi* for future applications (Tudzynski et al., 1996; Prado et al., 2004). Table 1 has listed the currently using transformants-screening markers for *F. fujikuroi.* Wiemann et al. (2012) knocked out the Sfp-Type 4′-Phosphopantetheinyl Transferase Ppt1. The resulted mutant strain became lysine auxotrophic and dramatically increased in GAs yield. It might be interesting to apply this strain for future lysine auxotrophic screening. Twaruschek et al. developed a plasmid that is able to recycle markers for continuous genetic engineering in *F. graminearum*, as such to overcome the shortage of selection markers in this species. In their strategy, the recombinase system Cre-loxP was activated upon induced expression to remove the marker genes after genetic engineering, while *URA3*/*pyrG* was involved in the system to counterselect marker removal isolates with 5-fluoroorotic acid (Twaruschek et al., 2018). Similarly, the yeast FLP recombinase has been applied in *Candida albicans* to recycle the nourseothricin resistance marker (Reuss et al., 2004), while a Cre-loxP marker recycling system has also been tested in *A. oryzae* (Mizutani et al., 2012). Induced expression of recombinases has been widely employed to recycle the selective makers, and in the meanwhile to remove the redundant DNA fragment after genetic engineering. This is a feasible strategy to get applied also in *F. fujikuroi*, especially when multiple genes need to be disrupted.

**TABLE 1:** Summary of the currently using markers for transformants screening in *F. fujikuroi*.

Type of selection		Marker gene	Example reference
Drug resistance markers	Nourseothricin resistance	*nat1*	Teichert et al., 2006; Janevska et al., 2017; Bomke et al., 2008
	Hygromycin resistance	*hph*	Studt et al., 2013a; Wagner et al., 2013
	Geneticin resistance	*nptII*	Castrillo et al., 2013
Nitrogen source	Nitrate dependent	*niaD*	Sanchez-Fernandez et al., 1991; Tudzynski et al., 1996; Prado et al., 2004

## Protoplast Preparation and Transformation Technologies

In many filamentous fungi, successful protoplast generation is the prerequisite of single cell isolation, efficient transformation, successful genetic engineering etc. *F. fujikuroi* is a polynuclear mycelial fungus. Some important industrial applying strains do not produce conidia, making it obliged to prepare protoplast for single cell isolation. Efficient cell wall degradation enzymes are of substantial importance to generate protoplasts. Some degradation enzymes, such as the snailase (Mink et al., 1990) and chitinase (Patil et al., 2013; Halder et al., 2014), have been frequently used to deconstruct the hyphae in some fungi. Shi et al. (2019) performed a series of optimizations on the preparation of protoplasts. This is the latest update about the optimized protocol for protoplast production in *F. fujikuroi*. They have tested five enzymes, including the lysozyme, snailase, cellulase, lysing enzyme and driselase. Only the lysing enzyme (Sigma-Aldrich, United States) and driselase (Sigma-Aldrich, United States) treated cells gave a reasonable amount of living protoplasts. The lysing enzyme and driselase were then tested in combination and the optimum ratio was obtained at 3:2 with a total concentration of 15 mg/L. Finally, the hydrolyzing time was optimized based on the amount of produced protoplast and cell regeneration efficiency, and the optimal hydrolysis time was ultimately chosen at 3.5 h.

There are many transformation methods available for *F. fujikuroi*. Electroporation, PEG (polyethylene glycol)-mediated transformation and *Agrobacterium* transformation are the three most popular transformation methods in filamentous fungi. The PEG-mediated transformation is an easy-to-operate method that usually combines a heat-shock process. This method has been frequently used to transform *F. fujikuroi* (Bruckner et al., 1992; Linnemannstons et al., 1999; Fernandez-Martin et al., 2000). Besides *F. fujikuroi*, PEG-mediated transformation method has also been frequently used in many other filamentous fungi, such as *Aspergillus fumigatus* (Fuller et al., 2015), Stagonospora nodorum (Liu and Friesen, 2012), and *Pseudogymnoascus verrucosus* (Diaz et al., 2019). Electroporation is another frequently used method for DNA transformation in fungi. This method is usually known to achieve high transformation efficiency. However, the applied voltage needs to be carefully adjusted, especially when the transformation is applied to the protoplast, a very week cell form. Garcia-Martinez et al. (2015) successfully implemented electroporation in *F. fujikuroi* at the voltage amplitude of 600 V/mm, with one pulse duration of 200 μs, in a cuvette with 1 mm electrode distance. The *Agrobacterium* transformation method has been successfully developed for the filamentous fungi for many years. It was claimed to be much more efficient than the conventional techniques (de Groot et al., 1998). In the *Agrobacterium* transformation protocol, both conidia and protoplast can be used as the host. This method has been succeed in transforming DNA in many different fungi, including *A. niger*, *Aspergillus awamori*, *Trichoderma rees*ei, *Colletotrichum gloeosporioides*, *Neurospora crassa* etc. The *Agrobacterium* transformation method has also been successfully applied in some *Fusarium* species, such as *F. oxysporum* (Islam et al., 2012), *Fusarium venenatum* (de Groot et al., 1998), and *F. proliferatum* (Bernardi-Wenzel et al., 2016). Zhu et al. (2009) tested the PEG and Agrobacterium-mediated transformations of a plasmid in *F. fujikuroi* using respectively protoplast and conidia as the competent cells. With their protocol, 15 transformants per μg of DNA and 37 transformants per 1 × 10^6^ conidia were harvest respectively by the PEG-mediated and Agrobacterium-mediated transformations.

## Genome Editing

### Homologous Recombination (HR)

Unlike the baker’s yeast, genome editing is usually inefficient in most of fungi. This is mainly due to the bad transformation efficiency and low HR rate. In *S. cerevisiae*, efficienct gene targeting can be carried out using 30–40 bp homologous flanking sequence on each side of the donor DNA, while in *F. fujikuroi* or many other fungi, we usually use 500 bp or longer homologous flanking sequences, even though the correct integration rate is still very low. We harvested 1 correct mutant after screening over 100 transformants when deleting a gene in *F. fujikuroi*, although a donor DNA construct harboring 700 bp homologous flanking sequence on each side was used (data not shown). In many other organisms, the “correct transformant rate” can be significantly improved by blocking the Non-Homologous End Joining (NHEJ) system. Usually, either gene *ku70/80* or gene *lig4* was knocked out to eliminate NHEJ in different fungi, including *N. crassa* (Ninomiya et al., 2004), *Kluyveromyces lactis* (Kooistra et al., 2004), *Cryptococcus neoformans* (Goins et al., 2006), *Aspergillus* spp. (da Silva et al., 2006; Takahashi et al., 2006; Meyer et al., 2007), *Pichia ciferrii* (Schorsch et al., 2009), and *Candida glabrata* (Ueno et al., 2007; Cen et al., 2015). In comparison to *KU70/80*, deletion of the *LIG4* gene showed to have less side effect, except for losing the NHEJ function (Daley et al., 2005; Schorsch et al., 2009; Cen et al., 2015). It will be interesting if we can delete the *lig4* gene in *F. fujikuroi*, as such to enhance the gene targeting efficiency in this species. However, according to our previous work, deficient NHEJ could not always make noticeably increase in HR efficiency, but reduce dramatically the ectopic integration rate (Cen et al., 2015). The CRISPR-cas system was frequently reported to promote the HR efficiency when compared to the classical approaches (Lin et al., 2014; Zhang Y. et al., 2016; Cen et al., 2017; Chung et al., 2017). Thus, applying the CRISPR-cas technology in an NHEJ deficient *F. fujikuroi* strain can be expected to ideally improve the genetic engineering efficiency. We suggest to apply the CRISPR-cas system in combination of *lig4* disruption (Cen et al., 2017).

### CRISPR-Cas

**FIGURE 3:**
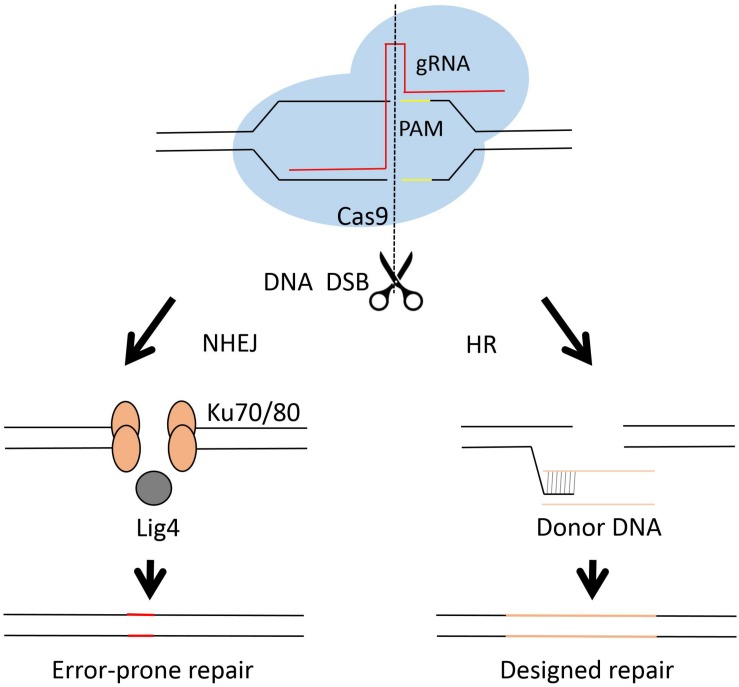
Diagram of Cas9 complex and DNA repair in genome engineering. The CRISPR-Cas system employs a short guide RNA to direct the Cas9 protein, an endonuclease, to a specific cutting site in the genome and generates DNA double strands break (DBS). The lethal DBS can be repaired by either NHEJ or HR. In the NHEJ process, proteins such as Ku70, Ku80 and Lig4 are involved. The NHEJ process results in error-prone repairs. In the HR process, usually a donor DNA is employed for precise genetic engineering.

The Clustered Regulatory Interspaced Short Palindromic Repeats (CRISPR) and CRISPR-associated (Cas) system has brought a remarkable development in genome engineering efficiency in different organisms during the past few years (Doudna and Charpentier, 2014; Albadri et al., 2017; Ermert et al., 2019; Pu et al., 2019; Song et al., 2019). Briefly, the CRISPR-cas system employs a guide RNA (gRNA) and an endonuclease, mostly a single nuclease Cas9 (Makarova et al., 2011; Chylinski et al., 2014), as the two working elements for a site directed DNA cutting. The cut DNA can be then repaired by either NHEJ or HR (see Figure 3). The NHEJ repair frequently caused unpredictable mutagenesis, while the homology directed repair may result in a seamless genetic editing. The first fungal CRISPR-Cas system was established in *S. cerevisiae* (DiCarlo et al., 2013). After that, the CRISPR-Cas system was developed very rapidly in different fungi, including *C. albicans* (Vyas et al., 2015), *C. glabrata* (Enkler et al., 2016; Cen et al., 2017), *C. neoformans* (Arras et al., 2016; Wang, 2018), *A. niger* (Zheng et al., 2018, 2019), *A. fumigatus* (Fuller et al., 2015), *A. oryzae* (Katayama et al., 2016) etc.

Although the CRISPR-cas technology has been used for a few years in filamentous fungi (Nodvig et al., 2015), this system was only tested successfully in *F. fujikuroi* very recently. Shi et al. (2019) developed a CRISPR-cas system for genome editing in *F. fujikuroi* as the first time at the beginning of 2019. In their work, three nuclear localization signal (NLS) peptides, the classical SV40 NLS, an endogenous histone NLS and an endogenous *Velvet* NLS, was tested to import the Cas9 protein into the nucleus. Finally, the NLS of native histone H2B was chosen to fuse with the Cas9 protein, as it gave the best mutagenesis rate. The promoter selection for gRNA transcription was the second challenge. Shi et al. (2019) evaluated three promoters, the polymerase II promoter, the endogenous polymerase III *U6* promoter and the endogenous 5SrRNA promoter, of which the 5SrRNA promoter gave the best editing efficiency. The resulted CRISPR-Cas system showed a genome editing efficiency of approximately 60–80%.

The advantage of this *F. fujikuroi* CRISPR-Cas system is that the expression of Cas9 protein did not show any effect to the cell growth, while the non-specific toxicity resulting from Cas9 expression or nuclease activity has been widely noticed in many other organisms (Morgens et al., 2016, 2017; Munoz et al., 2016; Cen et al., 2017). In some other microorganisms, such as *E. coli* and baker’s yeast, usually the CRISPR-Cas elements can be eliminated after the genetic manipulation, which not only avoided the possible toxicity of the Cas9 nuclease, but also enabled continuous genome editing. In *E. coli*, a dual-plasmid-based system was employed. The gRNA sequence was included in one plasmid (named as pTarget). The *cas9* gene, a temperature-sensitive replicon *repA101*(Ts) and an arabinose induced gRNA expression cassette that targets the pTarget plasmid were edited in another plasmid (named as pCas). The pTarget can be eliminated by adding arabinose, while the pCas can be removed by rising the temperature (Jiang et al., 2015). In *S. cerevisiae*, another dual-plasmid-based system was employed using a centromeric plasmid and a 2μ plasmid. In the centromeric plasmid, Cas9 can be expressed upon galactose induction. The gRNA containing 2μ plasmid can be eliminated by culturing the cells in non-selective medium (DiCarlo et al., 2013) for continuous genome editing. However, the current *F. fujikuroi* CRISPR-Cas system is a one-off system. For multi-gene editing, multiple gRNA expression cassettes have to be cloned into one plasmid to execute genetic engineering one time for all gene targets. The multi-gene disruption efficiency was also tested by Shi et al. The disruption efficiencies were 79.2, 10.8, and 4.2% for single, double and triple gene disruption, respectively. This result indicates that genetic manipulation for 3 genes or more would be very difficult in *F. fujikuroi* by this system.

Another type of CRISPR-Cas system has been developed for several non-*fujikuroi* fusaria, using the *in vitro* prepared Cas9 protein/gRNA ribonucleoproteins (RNPs). Transformation of *in vitro* prepared Cas9/gRNA RNPs has been claimed to be able to reduce the specific integration of the donor DNA, while the CRISPR elements can be degraded naturally after genetic manipulation (Wang et al., 2018). However, compared to the plasmid-transformation method, this RNPs-transformation method is more complicated in handling, as the Cas9 protein needs to be additionally purified and concentrated before the transformation. Ferrara et al. (2019) developed a CRISPR-Cas9 system for *F. proliferatum* by transforming *in vitro*-assembled dual Cas9 RNPs. Using this method, the genomic DNA was cut twice at a specific locus and the donor DNA can target the DSB (DNA double strand break) efficiently using a short homologous flanking sequence of 50 bp on each side (Ferrara et al., 2019). Such efficient HR (efficient DNA integration using 35-60 bp flanking arms) has also been achieved in some other filamentous fungi, including *Penicillium chrysogenum* (Pohl et al., 2016) and *A. fumigatus* (Zhang C. et al., 2016). The efficient homology directed repair may simplify the construction of donor DNAs and reduces off target rate. It is very interesting to also achieve it in *F. fujikuroi*.

## RNA-Mediated Gene Silencing

An alternative method to gene deletion is to silence gene expression at the post-transcriptional level, mostly known as RNAi (RNA interference). The RNAi technology has become an excellent tool to exploit gene function in microorganisms, plants and animals. Briefly, RNAi employs a specific double strand RNA and the homologous based mechanism to attack and degrade the targeted mRNA, and finally knockdown the gene expression. This technology has been successfully established in different filamentous fungi for many years (Liu et al., 2002; Goldoni et al., 2004; Mouyna et al., 2004; Ullan et al., 2008). McDonald et al. applied the RNAi technology in three filamentous fungal phytopathogens, two aspergilli and *F. graminearum*, over a decade ago (McDonald et al., 2005). More recently, Nino-Sanchez et al. developed an RNAi system for *F. oxysporum* on the basis of the dsRNA expression cassette used for *P. chrysogenum* and *Acremonium chrysogenum* (Nino-Sanchez et al., 2016). Compared to gene deletion, RNAi is apparently a better choice to target the essential genes. Unfortunately, to date there’s no report about RNAi application in *F. fujikuroi*. RNAi is highly conserved among many eukaryotes, thus has a great potential to be used also in *F. fujikuroi*.

## Fluorescent Protein Tags

### Bimolecular Fluorescence Complementation

Investigation of protein-protein interaction is essential for understanding the signal transduction and regulation of metabolism, and helps to reveal many intrinsic biological mechanisms. Bimolecular Fluorescence Complementation (BiFC) assay is a decent tool for *in vivo* observation of protein-protein interaction. Michielse et al. (2014) have applied the BiFC assay in *F. fujikuroi* several years ago. They tagged two transcription factors with two splitted gene fragments of an enhanced yellow fluorescent protein (EYFP). The two gene fragments encode the N terminal amino acids 1–154 and C terminal amino acids 155–238. Two transcription factors were then tagged with these two gene fragments and co-transformed into *F. fujikuroi*. The resulted transformant showed a co-localized fluorescence signal in the nucleus. This method was previously tested by Hoff et al. in another filamentous fungus (Hoff and Kuck, 2005), and was directly applied in *F. fujikuroi*. It would be more confident if we can test the BiFC assay systemically with additional controls to eliminate errors such as EYFP self-aggregation before we can start to apply it extensively in *F. fujikuroi*.

### Fluorescent Proteins

Fluorescent-protein tags are common tools to monitor the protein localization. It has been extensively practiced in the research of *F. fujikuroi* (Studt et al., 2013b; Pfannmuller et al., 2015). Michielse et al. (2014) tagged the GATA transcription factors AreA and AreB with GFP and RFP respectively to track their cytosolic or nucleic localization. Wiemann et al. (2010) tagged two proteins of a *velvet* complex, FfVel1 and FfLae1, by enhanced GFP and YFP, and visualized a nuclear co-localization signal. Garcia-Martinez et al. tagged the light-sensitive gene *carO* with enhanced YFP and visualized the membrane localization signal. To successfully express a functional fusion protein, they used an 18 bp DNA fragment to bridge the gene and tag (Garcia-Martinez et al., 2015).

## Quantification of Biomass

Biomass quantification techniques are very basic but important to monitor the cell proliferation. Usually, the microorganisms can be quantified simply by counting the cells using a hemocytometer, measuring the optical density, or weight the cell dry weight. Due to the filamentous nature of many fungi, dry weight measurement become the only feasible way to efficiently determine the biomass. However, *F. fujikuroi* is an industrial production microbe. Those traditional measurements cannot satisfy the complicate fermentation medium, which usually contains insoluble components, such as the corn/rice flour, soybean pulp and arachis flour. These components may form sticky paste like medium or present as insoluble particles. The tetrazolium salt (XTT) method is another efficient way to quantify the biomass. XTT can rapidly penetrate into the living cells and being catalyzed by the active dehydrogenase. Thus, the XTT method can also discriminate the active biomass from the cell debris and bio-inactive particles. The XTT reaction uses a color change for readout and has been applied in many filamentous fungi (Meletiadis et al., 2001; Antachopoulos et al., 2007; Moss et al., 2008). We developed an XTT assay to measure the active biomass of *F. fujikuroi* (Cen et al., 2018). The established method was then tested and approved using the industrial fermentation conditions. Using this method, the cell growth can be well monitored during the fermentation.

There are other methods available for biomass quantification, for instance, the ELISA method and the quantitative PCR (QPCR). These methods have a very high resolution, and are able to distinguish a trace amount of difference in biomass. Besides, they can also distinguish the targeted sample in a complicated mixture. As an example, both ELISA and QPCR were used to quantify the biomass of filamentous fungi in infected plants (Brunner et al., 2012; Song et al., 2014; Feckler et al., 2017). However, when the cell sample is in a large quantity, such as the cells in fermentation, then the big dilution factor could confer significant error to these assays.

## Fermentation Technologies

### Medium Composition

Gibberellin fermentation has a very long history since 1950s (Darken et al., 1959; Rodrigues et al., 2012). Development of fermentation technologies has been sustained for decades for the development of the gibberellin industry. The optimization of the fermentation conditions started since the early 1950s (Borrow et al., 1955). Darken et al. (1959) tested different carbon sources as the first time, and concluded that addition of slowly utilized carbon source may result in increased GAs production, while slow-feed of glucose in a fermentation also positively affected the GAs production. The carbon-catabolite-repression has been known for a long time, while addition of a large quantity of glucose inhibits GAs production. However, little is known about the molecular basis of this phenomenon. Based on the cost efficiency, the currently used industrial fermentation media are mostly composed of a large quantity of starch. Plant oils were also successfully used for gibberellin fermentations (Gancheva et al., 1984; Gokdere and Ates, 2014). The addition of plant oils was interpreted to inert the carbon-catabolite-repression (Tudzynski, 1999). However, it has not been experimentally verified. Addition of plant oils might also functions to balance the nutritional needs of the fungi and release the metabolic burden of alternative biosynthesis pathways. Besides, as a phytopathogen, it is reasonable that *F. fujikuroi* secretes GAs to infect plants in the case that nutrients are poorly present in nature. More complex carbon sources would possibly resemble the natural system better. Nitrogen inhibition has also been known for many years in gibberellin production (Borrow et al., 1964). Kuhr et al. (1961) investigated the influence of different nitrogen sources on the production of GAs. The complex organic nitrogen sources, such as the peanut meal, soybean meal and yeast extract, are favorable for the production of GAs. The molecular basis of nitrogen inhibition has been well studied during the past 10 years (Mihlan et al., 2003; Wagner et al., 2010, 2013; Michielse et al., 2014; Tudzynski, 2014). Other fermentation parameters, including the growth temperature, kinetics of nutrient metabolisms and impact of some other nutrients were also studied many years ago (Borrow et al., 1964; Bruckner et al., 1991). The plant extract seems to be in favor of, as they were frequently reported to promote the GA synthesis. Sucrose, corn steep liquor, glycerol, soybean pulp, arachis flour etc. were frequently chosen as the key components of fermentation media to promote GAs production, especially in the modern gibberellin industry (Cen et al., 2018). These fermentation factors, such the plant extract and temperature (25–28°C), are more like simulations of the nature environment, in which *F. fujikuroi* secretes GAs to invade the plant to survive and propagate. To date, modifications of fermentation conditions are still on going with the purpose to increase the productivity, reduce the cost and make it compatible to the following processings.

### Fermentation Types

Different types of fermentation have been established for GAs production. Currently, the most widely used industrial fermentation is the SMF. However, the SMF usually requires high energy consumption, is deficient in aeration, gets frequently contaminated and ends up with a large amount of waste water. The SSF is the most frequently tested fermentation other than SMF. The GAs yield is much higher in SSF in comparison to that of the other types of fermentation. The reported GAs yield reached more than 5 mg/g support after 7 days fermentation (Corona et al., 2005; Rodrigues et al., 2009; Satpute et al., 2010). The reason could be that SSF mimics best the growth conditions of this microorganism in nature, and is able to overcome all the previously mentioned shortcomings of SMF. In addition, the increased GAs yield might be largely due to the increased aeration. The supplied oxygen might be consumed during the GAs anabolism, as the monooxygenases play very important roles during the synthesis of GAs (Tudzynski, 1999, 2005; Albermann et al., 2013). Or probably the enhanced mitochondrial respiration benefited the cell growth in general and indirectly improved GAs synthesis. In an SSF system, the selection of support/substrate materials is crucial, as it may provide nutrients, serve as a support material, and induce product synthesis. Different types of substrate materials have been tested for GAs production, including wheat bran (Agosin et al., 1997; Bandelier et al., 1997), Coffee husk (Machado et al., 2002, 2004), citric pulp (Rodrigues et al., 2009; de Oliveira et al., 2017), Pigeon pea pods, Corncobs, Sorghum straw (Satpute et al., 2010) etc. The use of these plant derived materials/wastes reduced the production cost whereas all resulted in a high productivity of GAs. A variety of tested SSF experiments for GA_3_ production have been listed in Table 2. Water activity reflects the active part of moisture content, and is usually considered to correlate with microbial growth. It has been found that the water activity has a significant impact on GAs fermentation in a SSF. Usually, the water activity needs to reach 0.99 or higher for optimal cell growth and efficient GA production (Gelmi et al., 2002; Corona et al., 2005). However, unlike SMF, these SSF technologies are all non-conventional setup, and are currently only tested in the laboratory scale. The fermentation scale-up tests, further processings, such as the extraction and purification technologies, stay to be investigated before it can be applied in the industry. Oliveira et al. developed a semisolid state fermentation (SSSF) system using submerged citric pulp particles. They compared the SMF, SSF, and SSSF systems using similar citric pulp based media in both bubble columns (BCR) and Erlenmeyer flask reactors. In general, the GA productivity in BCRs is lower than that in the Erlenmeyer flasks, while the SSSF system is better than the SMF system in yield, indicating that an SSSF system could be possibly used to replace the SMF system while keep the current industrial facilities and production processes.

**TABLE 2:** Summary of various SSFs experiments for GA_3_ production.

Microorganism	Substrate	Productivity	Fermentation time	References
*Fusarium fujikuroi*	Wheat bran with soluble Starch	1.22 mg g^–1^	7 days	Kumar and Lonsane, 1986
*Fusarium moniliforme*	Maize flour mixed with wheat bran	19.3 mg g^–1^	18 days	Qian et al., 1994
*Fusarium fujikuroi*	Maize cob	4.8 mg g^–1^	22.9 days	Pastrana et al., 1995
*Fusarium fujikuroi*	Wheat bran	6.8 mg g^–1^	7.9 days	Agosin et al., 1997
*Fusarium fujikuroi*	Wheat bran	3 mg g^–1^	11 days	Bandelier et al., 1997
*Fusarium fujikuroi*	Coffee husk with cassava bagasse	0.4925 mg g^–1^	7 days	Machado et al., 2002
*Fusarium fujikuroi*	Coffee husk with cassava bagasse	0.925 mg g^–1^	6 days	Machado et al., 2004
*Fusarium fujikuroi*	Wheat bran and soluble starch	4.7 mg g^–1^	6 days	Corona et al., 2005
*Fusarium fujikuroi*	Cassava bagasse	1.58 mg g^–1^	8 days	Otálvaro et al., 2008
*Fusarium moniliforme*	Citric pulp with sucrose	5.9 mg g^–1^	3 days	Rodrigues et al., 2009
*Fusarium proliferatum*	Pigeon pea pod	7.8 mg g^–1^	8^–^10 days	Satpute et al., 2010
	Pea pods	6.4 mg g^–1^		
	Corncobs	6.1 mg g^–1^		
	Sorghum straw	5.5 mg g^–1^		
*Fusarium moniliforme*	Jatropha seed cake	225 mg g^–1^	6 days	Rangaswamy and Balu, 2010
*Fusarium moniliforme*	Jatropha seed cake	105 mg g^–1^	4 days	Rangaswamy, 2012
*Fusarium moniliforme*	Wheat bran with soluble starch	1.16 mg g^–1^	7 days	Desai and Panchal, 2016
*Fusarium fujikuroi*	Citric pulp	7.34 mg g^–1^	9 days	de Oliveira et al., 2017

## Strain Improvement for GA Production

*F. fujikuroi* is able to produce a variety of valuable secondary metabolites. There are at least over 10 different types of metabolites that are known to be produced by this microorganism. Forty seven putative gene clusters of different secondary metabolites have later been revealed and well documented (Wiemann et al., 2013; Janevska and Tudzynski, 2018). Among all these secondary metabolites, GAs are the iconic products, as they were discovered first, widely produced in industry, extensively applied in farming and with its genetic basis of metabolism best studied.

The excellent GAs yield is the trademark of *F. fujikuroi*. Besides optimization of the fermentation processes, strain improvement is also very crucial for industrial application. In industry, the researchers traditionally engaged in random mutagenesis and screening for improved production strains. These methods usually employ extreme physical conditions or toxic chemical reagents, and require an efficient screening protocol and a large amount of labor. An advantage over knock-out mutations is that these methods may generate unpredictable mutations on genes in question rather than just simply turn them off. This is probably the main reason why these methods are favored and widely practiced.

**FIGURE 4:**
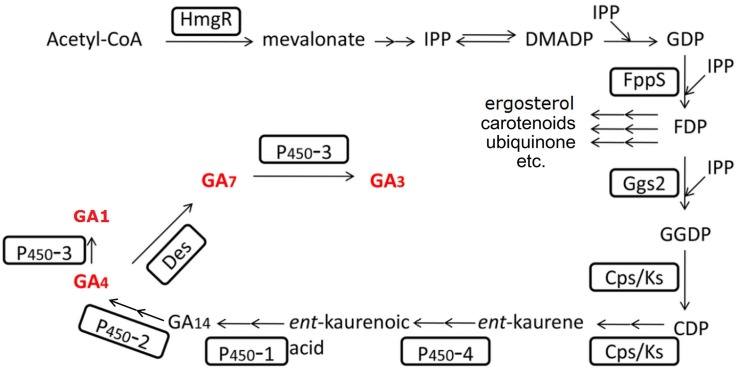
Gibberellin biosynthesis pathway in *F. fujikuroi*. The involved enzymes are highlighted by boxes; arrows indicate sequential biosynthesis steps (double arrows indicate multiple reactions); the rest names are either the main intermediates or end products. The bioactive GAs are marked in bold red letters.

The GAs synthesis pathway (see Figure 4) has been completely uncovered for about two decades (MacMillan, 1997; Tudzynski and Holter, 1998; Tudzynski, 1999; Hedden and Thomas, 2012). Many efforts have been contributed to improve the GA productivity by altering the metabolic pathway. Wiemann et al. deleted the *ppt1* gene, which is involved in the post-translational modification of some key enzymes of the non-gibberellin metabolic pathways. The mutated strain in the C-1995 strain background showed to have over twofold increased GAs yield, indicating that elimination of other metabolites synthesis might somehow reduce the metabolic burden and enhance the metabolic flow to GAs. However, this phenotype could not be reproduced in another *ppt1* mutant in the IMI58289 strain background (Wiemann et al., 2012). Albermann et al. (2013) overexpressed *ggs2*, the first gene of the gibberellin-specific pathway (see Figure 4), and resulted in an increased productivity of 50%. Interestingly, the increased productivity was due to the increased product of GA_4_/_7_, while the GA_3_ kept its original yield. Beside this, they overexpressed a truncated HmgR protein with the N terminal deleted. The N terminal of HmgR corresponds to the regulatory domain that involved in the inhibitory feedback to the protein. The constructed strain resulted in a 2.5-fold increase in GAs yield (Albermann et al., 2013). Compared to GA3, GA_4_, and GA_7_ may perform more excellently when treating different plants (Kim and Miller, 2009; Curry, 2012; Qian et al., 2018). Tudzynski et al. deleted the *P450-3* gene and the resulted mutant could not produce GA_1_ and GA_3_ anymore, while the production of GA_4_ and GA_7_ was significantly increased (Tudzynski et al., 2003). Shi et al. (2019) deleted the *P450-3* gene and harvested a strain with 4.6 times increase in GA_4_ and GA_7_ production. Based on this strain, they additionally overexpressed the */ks* genes and the truncated *hmgR* gene. The resulted strain showed to have increased production of GA_4_/GA_7_ mixtures of approximately eightfold (Shi et al., 2019).

To date, the genetic engineering works are mostly base on the GAs metabolic pathway. To engineer a gibberellin specific producer, genes that relate to the pathogenicity and other secondary metabolites might be redundant and will increase metabolic burden. Beside this, there is a great potential to reveal the upstream regulation network of gibberellin metabolism. Some unknown global regulators, such as the nutrient sensing and signaling pathways, might also be critical to GAs synthesis. Some of those regulators can certainly be the future targets to get genetically engineered for a supreme GA producer.

## Conclusion

*F. fujikuroi* has been designated as a prevalent plant pathogen for around one century, while as an industrial GAs producer for over 50 years. The research activities concerning its virulence, drug resistance, host-pathogen interaction, metabolic pathways, signal transductions, fermentation and strain improvement have been carried out for many years. However, there are still a lot unresolved puzzles on the biology of *F. fujikuroi*. The slow progress of biological research is largely due to the sluggish development of efficient molecular tools/technologies. In the recent years, with the increased research interests in this species, the toolkit for *F. fujikuroi* is expanding rapidly.

The optimized protoplast preparation and DNA transformation protocols, the established CRISPR-Cas system, the increased choices of promoters and selection markers etc. can satisfy now the basic needs for genetic manipulations. Challenges are also emerging. Firstly, it might be interesting to knock out the NHEJ machinery to increase the efficiency of transformants screening. The HR efficiency is relatively low and varies among different isolates of this species. In classic genome engineering, usually homologous flanking arms of 500–1500 bp are required for homology directed DNA integration. Even though, the editing efficiency was not ideal. Microhomology-mediated DNA integration using 35–60 bp homologous flanking arms has been achieved in many filamentous fungi with a CRISPR-Cas system. Optimization of the current CRISPR-Cas system is then necessary to enhance the HR efficiency for more efficient genome editing. Besides, it will be also interesting to construct a CRISPR-Cas system that is able to pop out the CRISPR-cas elements for continuous genetic manipulations. Secondly, investigation of large DNA fragment deletion is missing. A large amount of gene clusters of different secondary metabolites, secreted proteins and virulence genes are harbored in the *F. fujikuroi* genome. It would be interesting to develop a genome engineering tool to efficiently alter a large DNA fragment. Thirdly, RNAi should be tested in this microorganism to complement with the current genetic engineering methods, while development of episomal plasmids could be a valuable attempt in the research field.

There are many methods available for identification of the *Fusarium* species, such as the morphological identification, PCR methods and MALDI-TOF MS method. Among them, the morphological identification is less precise, while the MALDI-TOF MS is the most accurate and efficient with the fingerprints database of the FFSC established. However, MALDI-TOF MS requires a large facility, thus is not very popular at the current stage. The PCR methods were frequently used and also precise. This method took several conserved genes as the PCR targets. However, the amplified DNA fragments should always be necessary to be sequenced for confirmation. The *in vivo* plant infection assays have been tested extensively with *F. fujikuroi*, taking mostly the rice seedlings as the hosts. This assay can be efficiently implemented within 2 weeks and end up with different virulence parameters. Different fluorescent proteins (GFP, RFP, YFP) have been used to tag *F. fujikuroi* proteins. The BIFC assay was once tested to analyze protein-protein interaction. Further assessment of this technology is necessary, as some essential controls were missing. The FRET (fluorescence resonance energy transfer) technology is another important tool to analyze protein-protein interaction, whereas is presently missing for *F. fujikuroi* research.

Due to the economic importance of gibberellins, the fermentation technologies and strain improvement studies were better developed. However, the literatures of medium optimization are mostly from many years ago. The current industrial production conditions of SMF need to be updated. Other technologies, such as the SSF systems, were mostly carried out at the laboratory level. Further studies about the scale-up testes, system optimization and adaptation to post-fermentational processings remain to be carried out. The reported strain improvement works were mostly based on engineering of the GAs metabolic pathway. We lack knowledge about the upstream regulation/signal-transduction network. It will be also interesting to engineer a GAs production strain with the redundant pathogenesis genes and gene clusters of other secondary metabolites deleted.

## Author Contributions

Y-KC created the idea and wrote the first draft. J-GL participated in writing the section of CRISPR-Cas. Y-LW helped to correct Table 2. Y-LW and J-YW participated in writing the chapter of Fermentation technologies. Z-QL and Y-GZ finalized the draft.

## Conflict of Interest

The authors declare that the research was conducted in the absence of any commercial or financial relationships that could be construed as a potential conflict of interest.
